# Machine Learning Algorithms Introduce Evoked Potentials As Alternative Biomarkers for the Expanded Disability Status Scale Prognosis of Multiple Sclerosis Patients

**DOI:** 10.7759/cureus.80335

**Published:** 2025-03-10

**Authors:** Dionysia C Chrysanthakopoulou, Constantinos Koutsojannis

**Affiliations:** 1 Physiotherapy Department, School of Health Rehabilitation Sciences, University of Patras, Patras, GRC

**Keywords:** artificial intelligence, evoked potentials, expanded disability status scale, machine learning, multiple sclerosis

## Abstract

Machine learning (ML) has witnessed a notable increase in significance within the medical field, primarily due to the increasing availability of health-related data and the progressive enhancements in ML algorithms. Thus, ML can be utilized to formulate predictive models that aid in disease diagnosis, anticipate disease progression, tailor treatment to fulfill individual patient needs, and improve the operational efficiency of healthcare systems. Timely detection of a disease contributes to effective symptom management and guarantees that appropriate treatment is provided. In multiple sclerosis (MS), evoked potentials (EPs) show a strong correlation with the Expanded Disability Status Scale (EDSS), suggesting their potential as reliable predictors of disability progression. The aim of the present study is to apply artificial intelligence (AI) techniques to identify predictors linked to the progression of MS as assessed by the disability index (EDSS). It is essential to clarify the role of EPs in the prognostication of MS. We conducted an analysis of empirical data obtained from a medical database consisting of 125 records. Our primary objective is to construct an expert AI system capable of predicting the EDSS index through the application of advanced knowledge-mining algorithms. We have developed intelligent systems that predict the progression of MS utilizing ML algorithms, specifically decision trees and neural networks. In our experimental evaluation, decision trees, neural networks, and Bayes for EPs achieved accuracies of 88.9%, 92.9%, and 88.2% respectively, which are comparable to MRI that obtained accuracies of 88.2%, 96.0%, and 85.0%. The EPs can be established as predictors of MS with efficacy analogous to that of MRI findings. Further investigation is necessary to validate EPs, which are significantly less expensive, portable, and simpler to administer than MRI, as equally effective as imaging or biochemical methods in functioning as biomarkers for MS.

## Introduction

Multiple sclerosis (MS) is an autoimmune disease that affects the central nervous system (CNS) [1]. In MS, the immune system mistakenly attacks the protective myelin sheath that surrounds nerve fibers. It is characterized by inflammation, demyelination (the destruction of the protective myelin sheath surrounding nerve fibers), neuronal loss, and gliosis (scarring). This demyelination disrupts the normal flow of electrical impulses along the nerves between the brain and the rest of the body, resulting in a wide range of neurological symptoms. Symptoms can vary widely among patients with the most common being fatigue, difficulty walking, difficulty with coordination and balance, numbness or tingling, muscle weakness, vision problems (such as blurred or double vision), cognitive changes (including memory issues), and bladder and bowel dysfunction [2,3]. While there is no cure for MS, various treatments are available to manage symptoms, modify the disease course, and improve quality of life. Understanding MS is crucial for developing effective treatments and support systems for those affected by the disease. This disease is thought to involve a complex interplay of genetic, environmental, and lifestyle factors that trigger an abnormal immune response. This response leads to inflammation and damage to myelin, as well as neurodegeneration over time. From an epidemiology perspective, MS is more prevalent in women than men, with a female-to-male ratio of approximately 2:1 [4]. The disease typically affects individuals between the ages of 20 and 40. The prevalence of MS has been increasing, potentially due to better diagnostic techniques and an aging population. Moreover, integrating these findings into clinical practice could lead to a paradigm shift in how MS is diagnosed and monitored, ultimately improving patient outcomes through timely and targeted therapeutic interventions.

Pathology of multiple sclerosis

Regarding the pathology of MS, recent findings have significantly advanced the understanding of the roles of B cells and microglia [5]. To elaborate, B cells are now recognized as active participants in the inflammatory processes of MS. They can infiltrate the CNS and contribute to the formation of inflammatory lesions. Their presence in the meninges correlates with the degree of cortical lesions and neurodegeneration, as well as clinical disability. B cells can act as antigen-presenting cells, which means they can present antigens to T cells, thereby influencing T cell activation and the overall immune response [6]. This interaction is crucial in the development and progression of MS. Moreover, studies have shown that B cells can secrete factors that are cytotoxic to oligodendrocytes, the cells responsible for myelin production [7]. This cytotoxicity may contribute to the demyelination observed in MS. B cell-depleting therapies, such as monoclonal antibodies targeting CD2, have emerged as effective disease-modifying treatments (DMTs) for MS. These therapies aim to reduce the number of pathogenic B cells and modulate the immune response. In summary, T helper cells are central to the autoimmune processes in MS, driving inflammation and myelin damage, which ultimately leads to the neurological symptoms characteristic of the disease.

As for microglia, the resident immune cells of the CNS play a dual role in MS pathology. They can become activated in response to injury or inflammation and contribute to both protective and detrimental processes [8]. Activated microglia can release pro-inflammatory cytokines that exacerbate neuroinflammation. Recent research by Yong in 2022 suggests that microglia are involved in the neurodegenerative aspects of MS [9]. They can contribute to neuronal damage and loss, particularly in progressive forms of the disease. The loss of "homeostatic" microglia has been associated with patterns of activation that correlate with disease severity. Targeting microglial activation and promoting their protective functions could be a strategy for managing MS. Overall, the evolving understanding of B cells and microglia highlights their critical roles in both the inflammatory and neurodegenerative components of MS.

Diagnosing multiple sclerosis

As far as diagnosis, it typically involves clinical evaluation, MRI scans to identify lesions, and sometimes lumbar puncture to analyze cerebrospinal fluid (CSF) [10]. Using a combination of measures to obtain a standardized and objective assessment of MS is essential for personalized management and long-term monitoring of the disease. Some of the key clinical outcome measures in MS include the following: starting with the Expanded Disability Status Scale (EDSS), which is a widely used physician-based measure that assesses disability in MS patients based on neurological examination findings [11]. The EDSS is well-established and widely recognized in clinical practice and research. It provides a rapid assessment of disability status based on a neurological examination, making it a practical tool for clinicians. Nevertheless, the EDSS is an ordinal scale that ranges from 0 (normal neurological function) to 10 (death due to MS). This scale allows for a straightforward categorization of disability levels, but it is nonlinear, which can complicate the interpretation of changes over time. Another measure is the patient-determined disease steps (PDDS), which is a patient-reported outcome (PRO) measure that allows individuals with MS to self-assess their level of disability [12]. Also, there is the Neurologic Rating Scale (NRS), a scale that is used to evaluate neurological impairment and disease progression in MS patients [13]. In addition, Functional Systems Score (FSS) assesses various functional systems affected by MS, providing a comprehensive view of the patient's condition [14]. Finally, PROs are measures that capture the patient's perspective on their health status, symptoms, and quality of life, which are crucial for understanding the impact of MS from the patient's viewpoint [15].

MS is a heterogeneous disease, and reliable clinical outcome measures help capture the variability in symptoms and disability among patients [16]. This understanding is crucial for developing personalized treatment approaches and for recognizing the diverse experiences of individuals living with MS.

Identifying biomarkers for multiple sclerosis progress

In more detail regarding biomarkers, the biomarker Myelin Basic Protein (MBP) is correlated with EDSS scores and is found in higher levels during acute exacerbations of MS compared to remission and slower progressive forms of the disease. Also, the Myelin Oligodendrocyte Glycoprotein (MOG) biomarker is associated with distinct myeloid cell types in subjects with neuroinflammation and is used for diagnostic purposes in certain cases of MS. Moreover, plasma and CSF biomarkers related to inflammation, such as interleukin-6, have been studied in relation to MS [17]. Future research in MS should be directed toward a multi-faceted approach that combines technological advances and immunological insights. Combining various biomarkers, such as serum neurofilament light chain (sNfL) levels with other proteomic markers, could enhance prognostic accuracy in MS. This approach may help in better-defining relapses and disease progression. In addition, the use of advanced multiplex proteomic assays is recommended to identify panels of serum proteins that can provide more comprehensive insights into MS than single biomarkers alone. Another option should be the identification of non-invasive biomarkers. This work could be a call for the discovery of non-invasive, safe, and easily detectable biomarkers that can reliably predict disease activity and progression, which would be beneficial for clinical trials and patient management [18]. Today, while MRI is considered one of the primary tools for diagnosing MS, it is essential to examine a broader range of biomarkers, such as somatosensory evoked potentials (SEPs) [17]. These additional biomarkers are crucial not only for diagnosis but also for providing prognostic insights into the current condition. Therefore, it is necessary to develop a comprehensive set of neurophysiological examinations to enhance the reliability of early MS identification.

Classification in multiple sclerosis

MS can occur in different types. To start with, there is RRMS, which is characterized by episodes of new or worsening symptoms (relapses) followed by periods of recovery (remissions) [1]. Then, there is primary progressive multiple sclerosis (PPMS) that involves a gradual worsening of symptoms from the onset without distinct relapses or remissions. Finally, secondary progressive multiple sclerosis (SPMS) initially starts as RRMS but later transitions to a phase of progressive decline. There is often significant overlap between different clinical phenotypes of MS, such as RRMS and SPMS [19]. This overlap can make it difficult to categorize patients accurately, as individuals may exhibit characteristics of more than one phenotype over time. Furthermore, there is a transition between phases. The transition from RRMS to SPMS is not always clear-cut, and the timing and nature of this transition can vary widely among patients. This ambiguity complicates the classification process, as it can be challenging to determine the appropriate phenotype at any given time. Also, there is no single clinical, imaging, or laboratory characteristic that can definitively differentiate between MS subtypes. While certain markers such as neurofilament levels and lesion formation rates may show relative differences, they do not provide a clear distinction, leading to reliance on clinical characteristics for classification. Additionally, issues occur with subjectivity in historical reporting because classification often depends on patients' recollections and descriptions of their historical disease course, which can be subjective and variable. This reliance on patient history can introduce inconsistencies in how phenotypes are classified. These challenges highlight the need for ongoing research to develop more accurate biomarkers and classification systems that can better capture the complexity of MS and improve patient management and rehabilitation [20].

Risk factors for multiple sclerosis

Looking deeper into key genetic risk factors associated with MS, it has been found that the strongest genetic association with MS is in the human leukocyte antigen (HLA) region, particularly the HLA-DRB1 gene. Certain alleles, such as HLA-DRB1*15:01, are linked to an increased risk of developing MS [21]. This gene plays a crucial role in the immune system by presenting antigens to T cells, influencing immune responses. In addition to HLA-DRB1, several other non-HLA genetic variants have been identified through genome-wide association studies (GWAS). These include genes involved in immune regulation, such as IL2RA (interleukin 2 receptor alpha), and genes related to the function of the CNS, such as those involved in myelin formation and repair.

Regarding family history, having one increases the risk of developing the disease [1]. First-degree relatives of individuals with MS have a higher likelihood of being diagnosed compared to the general population, indicating a genetic predisposition. Also, MS is more common in individuals of Northern European descent, suggesting that specific genetic backgrounds may contribute to susceptibility. Moreover, MS is associated with interactions between environmental factors [1]. Genetic predisposition to MS is thought to interact with environmental factors, such as vitamin D levels, smoking, and viral infections (e.g., Epstein-Barr virus), which may further influence the risk of developing the disease. Low levels of vitamin D have been consistently associated with an increased risk of developing MS [22]. Vitamin D is thought to have immunomodulatory effects, which may help regulate the immune system and reduce the risk of autoimmune diseases like MS. MS prevalence is higher in regions farther from the equator, where sunlight exposure, and consequently vitamin D synthesis, is lower. This geographic pattern suggests that insufficient sunlight exposure may contribute to the risk of MS. While the role of vitamin D supplementation in preventing MS is still being studied, maintaining adequate vitamin D levels is often recommended for individuals at risk of or diagnosed with MS, with suggested target levels of 40-60 ng/mL.

As for obesity, studies have shown that obesity during childhood and adolescence is associated with an increased risk of developing MS later in life [1]. This association may be due to the inflammatory effects of excess body fat, which can influence immune function. In addition to increasing the risk of developing MS, abdominal obesity has been linked to worse disability outcomes in individuals already diagnosed with MS. This suggests that obesity may not only be a risk factor but also a contributor to disease progression. The mechanisms by which obesity affects MS risk may include increased systemic inflammation, altered immune responses, and changes in metabolic health, all of which can impact the pathogenesis of autoimmune diseases.

Treatment in multiple sclerosis

The treatment plan for MS varies based on the specific symptoms experienced by the patient and the type of MS they have. Disease-modifying therapies (DMTs) are used to reduce the frequency and severity of relapses and slow disease progression. DMTs are particularly important for patients with relapsing forms of MS, like RRMS and SPMS, and may include medications such as interferons, glatiramer acetate, and newer oral therapies [23]. For acute relapses, corticosteroids, such as methylprednisolone, are commonly prescribed to reduce inflammation and speed recovery. In addition, various medications and therapies are used to manage specific symptoms of MS, including spasticity, urinary incontinence, bowel dysfunction, fatigue and cognitive dysfunction, and psychological support. Regarding spasticity, treatment may involve physiotherapy and medications such as baclofen or gabapentin. If these are insufficient, secondary options like tizanidine or dantrolene may be considered, and benzodiazepines can be used as a third-line treatment [23]. Additionally, for urinary incontinence, anticholinergic medications and tricyclic antidepressants are options, along with intermittent self-catheterization for severe cases. As for bowel dysfunction, management may include stool softeners and dietary changes to increase fiber intake. Symptoms like fatigue and cognitive dysfunction may require a combination of lifestyle modifications, cognitive therapies, and sometimes medications to help manage [24]. Last but not least, given the emotional and psychological impact of MS, treatments may also include counseling and support for mental health issues such as depression and anxiety [25].

Physiotherapy in Multiple Sclerosis Rehabilitation Strategy

Physiotherapy is a huge and necessary part of the management of MS by addressing various symptoms and improving the overall quality of life for individuals with the condition. As mentioned earlier, in regard to the management of spasticity, physiotherapy interventions, such as exercise therapy and electrical stimulation, have been shown to effectively reduce spasticity in individuals with MS [26]. These interventions can help improve muscle tone and reduce discomfort associated with spasticity. Physiotherapists design tailored exercise programs that focus on enhancing mobility, strength, and endurance. This is particularly important for individuals with MS, as mobility challenges are common. Techniques such as gait training and balance exercises can significantly improve functional abilities. Additionally, exercise therapy is beneficial in managing fatigue, a common symptom in MS. Structured physical activity can help increase energy levels and reduce the perception of fatigue. By improving physical function and reducing symptoms, physiotherapy can enhance the overall quality of life for individuals with MS. This includes promoting independence in daily activities and improving psychological well-being. According to Etoom et al. [26], the most effective physiotherapy interventions for spasticity in people with MS were outpatient exercise training, which was particularly beneficial for individuals with stable MS; robot-assisted gait training and body weight-supported treadmill training, which improved gait and mobility; electrical stimulation, which provided relief and improved muscle function; therapeutic exercises, including strength and flexibility training; and core stabilization exercises, which have been shown to significantly improve muscle tone, balance, and walking ability, thereby addressing functional impairments associated with spasticity. Furthermore, findings based on another research support the importance of structured rehabilitation programs tailored to improve mobility and balance in people with MS, highlighting the need for specific and adequately dosed interventions [27]. Intensive training is linked to long-term potentiation and an increase in synapses within the motor cortex, enhancing motor output and functional recovery. The dose-response relationship is crucial in determining the effectiveness of rehabilitation interventions, with higher intensity, longer duration, and increased frequency of sessions leading to better outcomes in mobility and balance for individuals with MS. Physiotherapy is often part of a multidisciplinary team approach in MS management, collaborating with other healthcare professionals to provide comprehensive care tailored to the individual's needs [28].

Prognosis in multiple sclerosis

The prognosis of MS varies significantly among patients due to several factors, including the type of MS, individual disease characteristics, and response to treatment [29]. One of the aspects influencing prognosis is the disease type. Patients with clinically isolated syndrome (CIS) or relapsing-remitting multiple sclerosis (RRMS) often have a better prognosis compared to those with PPMS [30]. RRMS typically allows for periods of remission, while PPMS is characterized by a gradual progression of disability without clear relapses. Other aspects affecting the prognosis are the clinical and imaging markers. Various clinical, imaging, and laboratory markers have been identified as prognostic factors. For instance, a higher baseline MRI lesion load in patients with CIS is associated with an increased risk of developing MS and accumulating disability over time. The presence of specific MRI findings, such as the number and location of lesions, can also influence prognosis.

About the response to treatment, the effectiveness of DMTs can vary among patients, impacting long-term outcomes [19]. Patients who respond well to treatment may experience slower progression of the disease and a better overall prognosis. There is considerable variability in disease activity and progression among individuals, making it challenging to predict outcomes accurately. Factors such as age at onset, gender, and genetic predispositions may also play a role in determining the course of the disease.

Evoked potentials in multiple sclerosis

Evoked potentials (EPs) are valuable tools for diagnosing and managing MS [31]. They can help identify the type of neurological damage, such as demyelination or axonal degeneration. EPs are non-invasive and can track changes in the CNS. Additionally, they are unique as they directly assess the physiological changes associated with MS. This information can help to determine the disease's location, type, and progression. They are particularly useful for monitoring spinal cord damage as well. Using Virtual EPs and SSEPs in early evaluations of patients with CIS is also recommended [31]. Early diagnosis allows prompt treatment with DMTs. Periodic EPs can help monitor disease progression and assess physiological status. This is especially important for spinal cord disease, which might not be as easily detected on imaging.

EPs can serve as valuable prognostic and response biomarkers in clinical trials, particularly in assessing remyelination therapies and monitoring MS progression [32]. The combination of different EP modalities into a multimodal score showed closer associations with global clinical measures, enhancing the ability to assess the overall impact of therapeutic interventions. Multimodal evoked potentials (MEP) refer to the combined assessment of different types of EPs to provide a comprehensive evaluation of the CNS's function. In the context of MS, MEPs usually include visual evoked potentials (VEPs), which measure the electrical activity in the brain in response to visual stimuli and are particularly sensitive to pathology in the optic nerve; somatosensory evoked potentials (SSEPs), which assess the electrical activity in response to sensory stimuli applied to the skin, reflecting the function of the somatosensory pathways; and motor evoked potentials (MEPs), which evaluate the electrical activity in response to stimulation of the motor pathways, providing insights into the integrity of the pyramidal system. MEPs are primarily markers of the integrity of the corticospinal tract, which is crucial for motor function [33]. The importance of assessing these potentials to understand the extent of motor pathway involvement in MS is significant. MEPs have been included in EP scales that correlate well with clinical disability, as measured by the EDSS [34]. They are used in clinical trials to monitor the effects of DMTs and to assess neurodegeneration and axonal loss early in the disease process. This makes MEPs a promising tool for evaluating treatment efficacy and disease progression in MS. By integrating data from these different modalities, MEPs can offer a more comprehensive picture of the functional status of various neural pathways affected by MS. This approach could enhance the sensitivity and specificity of assessments, allowing for better monitoring of disease progression and treatment response as they have also shown utility in both diagnosis and prognosis of MS [32].

Quantitative EP scores demonstrate higher sensitivity to change compared to traditional clinical assessments in monitoring MS [35,36]. Specifically, quantitative EP scores can detect improvements or progression in the disease more effectively than ordinal EP scores or clinical evaluations. This enhanced sensitivity makes quantitative EP scores particularly suited for longitudinal studies and for assessing the efficacy of treatments over time. According to Hardmeier and Fuhr [32], several pieces of evidence support the use of EP as biomarkers in clinical trials for MS because of their prognostic value, their sensitivity to change, and their response to treatment. In addition, the prognostic value of EPs can be enhanced when combined with other biomarkers, such as MRI findings. These findings collectively support the rationale for using EPs as candidate biomarkers in clinical trials aimed at testing new therapies for MS, particularly those targeting remyelination and halting disease progression. The prognostic value of EPs lies in their ability to provide objective, sensitive, and predictive information about disease progression in MS, making them a valuable tool for both clinical practice and research in understanding the disease's trajectory.

Artificial intelligence technological approaches

The ongoing advancement of technology has facilitated the emergence of novel methodologies predicated on the principles of artificial intelligence (AI) and machine learning (ML). The escalation of health-related issues has resulted in a corresponding proliferation of big data. The effective utilization of such data necessitates the establishment of an automated system for disease forecasting, which entails the creation of ML algorithms that can operate proficiently in the face of potential challenges inherent in the datasets. AI represents a domain within computer science that encompasses the emulation of human cognitive capabilities utilizing computational mechanisms [37]. AI instruments are predicated on expert systems and algorithms, enabling the classification, interpretation, and synthesis of guidance and elucidations concerning the amassed data. Although AI is predominantly associated with computer science, it concurrently intersects with a myriad of scientific disciplines, including mathematics, cognitive science, philosophy, psychology, and biology, and has recently found integration within the engineering sector. ML employs statistical techniques such as regression analysis and Bayesian inference to forecast the categorization of data subjects derived from a dataset. It utilizes methodologies such as thresholding (pertaining to images), feature extraction, and pattern identification in addition to employing statistical models for predictive purposes. This domain cultivates learning paradigms such as supervised learning and unsupervised learning. Supervised learning is characterized as a methodological approach wherein the model is instructed and utilizes novel data to forecast outcomes. Conversely, in unsupervised learning, the algorithm constructs a model based on a specified set of inputs in the form of observations without prior knowledge of the anticipated outputs (clustering), which can uncover novel patterns within data by inputting training datasets devoid of human interpretations.

The incorporation of neural networks within the medical domain has been profoundly impactful. These networks excel in resolving highly intricate problems where traditional algorithmic methods prove inadequate or excessively convoluted. Their successful application in the medical field spans various domains, including drug development, patient diagnosis, and image processing. They contribute significantly to critical areas such as the identification of coronary artery disease and the analysis of electroencephalography (EEG) signals. They provide enhanced capacities for data analysis, pattern recognition, and decision-making, thereby driving advancements in medical research and clinical practices [38]. For instance, a systematic review published in the *Journal of the National Cancer Institute* assessed the external validation of AI algorithms for automated interpretation of screening mammography. The review found that most studies demonstrated incremental diagnostic accuracy improvements over radiologist interpretation alone, highlighting AI's potential to enhance diagnostic processes in medical imaging [39]. Additionally, another comprehensive review highlighted AI's superior accuracy in cancer diagnosis and prognosis prediction compared to traditional statistical methods. The integration of ML and deep learning techniques has led to unprecedented performance in predicting cancer outcomes, facilitating early interventions and personalized treatment strategies [40].

The main goal of this work is to create an AI system that predicts the progression of EDSS. The rest of the paper is organized as follows: The second section describes the Materials and Methods. The third section presents the Results. The fourth section provides a summary, as well as the future work we aim to achieve.

## Materials and methods

The dataset used in this study is available in the Kaggle Repository (https://www.kaggle.com/code/desalegngeb/predictors-of-multiple-sclerosis-disease). This dataset is the result of a prospective cohort study conducted on Mexican mestizo patients newly diagnosed with MS, who presented at the National Institute of Neurology and Neurosurgery (NINN) in Mexico City, Mexico, between 2006 and 2010. Data were collected in a study conducted on Mexican mestizo patients newly diagnosed with MS, who presented at the INNN in Mexico City, Mexico, between 2006 and 2010 [41]. The study aimed to evaluate the clinical, electrophysiological, and imaging characteristics of these patients, as well as their long-term outcomes and potential risk factors for the progression of MS. The findings revealed significant correlations between specific clinical features and the likelihood of progression, highlighting the importance of early intervention and monitoring in this patient population. The dataset includes 16 input parameters (including clinical and biochemical findings, MEPs, and multimodal MRIs) and two output EDSS scores, initial and final (Table 1). Some of the technical/medical terms are defined in Table 2.

**Table 1:** Parameters. ULSEP, upper limb somatosensory evoked potential; LLSEP, lower limb somatosensory evoked potential; EDSS, Expanded Disability Status Scale; BAEP, brainstem auditory evoked potential; VEP, visual evoked potential; SEP, somatosensory evoked potentials

Parameters	Specifics
Age	Age of the patient (in years)
Schooling	Time the patient spent in school (in years)
Gender	1=male, 2=female
Breastfeeding	1=yes, 2=no, 3=unknown
Varicella	1=positive, 2=negative, 3=unknown
Initial symptoms	1=visual, 2=sensory, 3=motor, 4=other, 5= visual and sensory, 6=visual and motor, 7=visual and others, 8=sensory and motor, 9=sensory and other, 10=motor and other, 11=visual, sensory and motor, 12=visual, sensory and other, 13=visual, motor, and other, 14=sensory, motor, and other, 15=visual, sensory, motor, and other
Mono or polysymptomatic	1=monosymptomatic, 2=polysymptomatic, 3=unknown
Oligoclonal bands	0=negative, 1=positive, 2=unknown
LLSEP	0=negative, 1=positive
ULSEP	0=negative, 1=positive
VEP	0=negative, 1=positive
BAEP	0=negative, 1=positive
Periventricular MRI	0=negative, 1=positive
Cortical MRI	0=negative, 1=positive
Infratentorial MRI	0=negative, 1=positive
Spinal cord MRI	0=negative, 1=positive
Initial EDSS	index at the onset of the disease
Final EDSS	index after the disease progress

**Table 2:** Medical terms that are referred to as parameters. VZV, varicella zoster virus; BAERs, brainstem auditory evoked responses; BAEPs, brainstem auditory evoked potentials; VEP, visual evoked potential; SEP, somatosensory evoked potential; LLSEP, lower limb somatosensory evoked potential; EDSS, Expanded Disability Status Scale; MS, multiple sclerosis; CNS, central nervous system; OCBs, oligoclonal bands; CSF, cerebrospinal fluid

Terms	Definitions
Varicella	Another name for chickenpox, or chicken pox, is a highly contagious disease caused by the initial infection with the VZV, a member of the herpesvirus family.
BAEP	In human neuroanatomy, BAEPs, also called BAERs, are very small auditory evoked potentials in response to an auditory stimulus, which are recorded by electrodes placed on the scalp.
VEP	VEP is an evoked potential elicited by presenting a light flash or pattern stimulus, which can be used to confirm damage to the visual pathways including the retina, optic nerve, optic chiasm, optic radiations, and occipital cortex.
SEP	SEP are recorded from the CNS following stimulation of peripheral nerves. ULSEP, LLSEP.
EDSS	The EDSS is a method of quantifying disability in MS and monitoring changes in the level of disability over time. It is widely used in clinical trials and in the assessment of people with MS [11].
OCBs	OCBs are bands of immunoglobulins that are seen when a patient's blood serum or CSF is analyzed. They are used in the diagnosis of various neurological and blood diseases. Oligoclonal bands are present in the CSF of more than 95% of patients with clinically definite MS.

The purpose of the study was to identify what symptoms/factors are better predictors of MS using ML approaches. In our work, the importance of EP recordings were examined among all other input parameters for initial as well as final EDSS scores.

## Results

The training set, randomly selected as 66% of the total, was used to train the ML models, while the testing set was used to evaluate the performance of the models produced by the Waikato Environment for Knowledge Analysis (WEKA). The subsequent ML models employed in this investigation comprised decision trees (J48), artificial neural networks (ANNs) (multilayer perceptron), and Bayes (Naïve Bayes) algorithms. The models were executed utilizing the WEKA platform [42]. The WEKA represents a widely utilized software suite for ML, developed in the Java programming language at the University of Waikato, New Zealand. This software is distributed as free software under the GNU General Public License. WEKA 3.7.8 encompasses a suite that includes a diverse array of visualization tools and algorithms tailored for data analysis and predictive modeling, in conjunction with graphical user interfaces that facilitate user accessibility to these functionalities. It incorporates data pre-processing capabilities implemented in C, as well as a Makefile-based system designed for the execution of ML experiments. The platform integrates various AI methodologies and statistical techniques. WEKA is conducive to several fundamental data mining processes, particularly data pre-processing, grouping, sorting, regression, clustering, visualization, and rule selection. All methodologies within WEKA are predicated on the premise that data is presented as a singular file or correlation, wherein each data point is characterized by a predetermined number of attributes (normal, numerical, or nominal, with additional attribute types also being supported). The WEKA software employs standard mathematical algorithms for ML, many of which culminate in decision trees for the purpose of data classification.

The first ML approach that was used is the decision trees with the J48 algorithm [43]. Through the utilization of decision trees, one can discern significant information, thereby enabling the construction of predictive models. A decision tree functions as a flowchart that systematically partitions the data into branches, ensuring that no data is lost while facilitating a sequence of decision-making processes. It serves as a sorting tree that functions as a navigational tool for making predictions based on a sequence of decisions informed by input data. The methodology for constructing the tree is as follows: each branch of the tree represents a decision point, where a choice is made based on the input of a single parameter to proceed to the next branch. This process is repeated until the leaf node reaches the predicted outcome (the EDSS prediction). Constructing the decision tree requires an immediate step to evaluate the model's accuracy. We employed the random tree algorithm to generate the decision tree based on our data.

As a second ML approach, WEKA's neural network (NN) was utilized to establish an ANN, which is classified under supervised ML and is recognized as one of the most prevalent methodologies in the medical domain [44]. For the implementation of this intelligent system, we employed WEKA’s multilayer perceptron, which includes a hidden layer. The algorithm employed in the NN is the error back-propagation algorithm. More specifically, our NN was structured in the form of I - H - O (input - hidden layers - output layers). We commenced the training of the NN by incrementally increasing the number of neurons (H) within the hidden layer and gradually augmenting the training epochs. By maintaining a constant learning rate, we observed a progressive decline in the error per training epoch, accompanied by a consistent enhancement in the classification results. Consequently, with seven hidden neurons and 15,000 training epochs, we achieved the optimal outcome. Beyond this point, the performance of the NN demonstrated stability.

**Figure 1:**
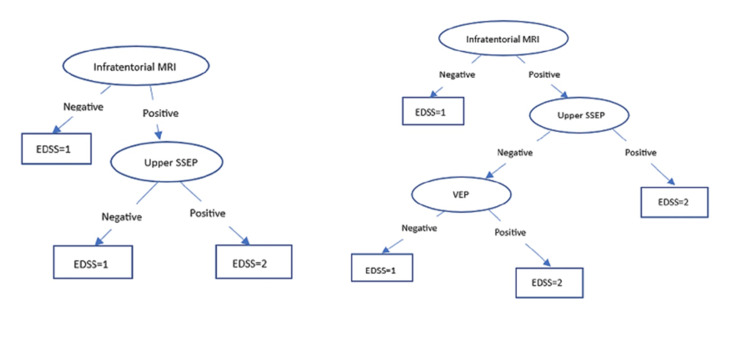
Decision tree examples resulting from the J48 algorithm.

Finally, Bayesian classifiers were employed, which constitute a compendium of classification algorithms grounded in Bayes' theorem. Rather than being a singular algorithm, it represents a family of algorithms, all of which adhere to a foundational principle: that each pair of features undergoing classification operates independently of one another. To commence our discussion, let us examine a dataset. Among the most straightforward yet efficacious classification algorithms is the Naïve Bayes classifier, which facilitates the expedited development of ML models endowed with swift prediction capabilities [45,46]. The Naïve Bayes algorithm is predominantly utilized for classification tasks. Its application is particularly prevalent in text classification endeavors. In tasks involving text classification, the data is characterized by high dimensionality, given that each individual word corresponds to a distinct feature within the dataset. It finds utility in domains such as spam filtering, sentiment analysis, and rating classification, among others. A salient advantage of employing the Naïve Bayes classifier is its operational speed. It exhibits rapid processing capabilities and simplifies the prediction process, even in the context of high-dimensional data. This model estimates the likelihood that an instance (such as various types of EPs) belongs to a specific class based on a defined set of feature values (final EDSS). It functions as a probabilistic classifier, predicated on the assumption that the presence of one feature in the model is independent of the existence of any other feature. In other terms, each feature contributes to the predictive outcomes without interdependencies. In practical applications, this condition is seldom satisfied. The algorithm employs Bayes' theorem as a foundational component for both training and prediction. Below are some examples using WEKA (Figure 1).

The performance of the ML models was evaluated using accuracy, precision, recall, F1 score, and the area under the receiver operating characteristic curve (AUC-ROC) on the test set (the remaining 34% of the total, randomly selected). The results of the models on the test set are shown in Figure 1 and Table 3. We calculated the prediction accuracy for disease diagnosis, the final EDSS score after disease progression, and disease follow-up based on all input parameters. Possible biomarkers were categorized into EPs (with a specific analysis of upper limb EP performance), MRIs (with a specific analysis of cortical MRI performance), and all other biochemical data.

**Table 3:** Accuracy results for different biomarkers prediction (%). EDSS, Expanded Disability Status Scale

Decision trees (J48)	All	EPs	MRIs	Upper EP	Cortical MRI	Other
Initial EDSS	89.3	82.5	81.1	85.0	85.8	85.8
Final EDSS	94.5	85.8	82.7	82.7	82.6	81.8
Disease follow-up	88.1	88.9	88.2	85.8	85.8	85.8

Neural networks (multilayer perceptron)						
Initial EDSS	95.3	95.3	97.5	90.6	96.8	85.8
Final EDSS	96.0	98.4	94.5	92.9	97.6	91.3
Disease follow-up	94.5	92.9	96.0	94.5	96.6	94.5

Bayes (Naïve Bayes)						
Initial EDSS	72.0	71.7	68.5	70.1	70.0	66.9
Final EDSS	78.4	67.7	75.6	66.9	65.4	63.8
Disease follow-up	85.0	88.2	85.0	86.6	86.6	86.6

**Figure 2:**
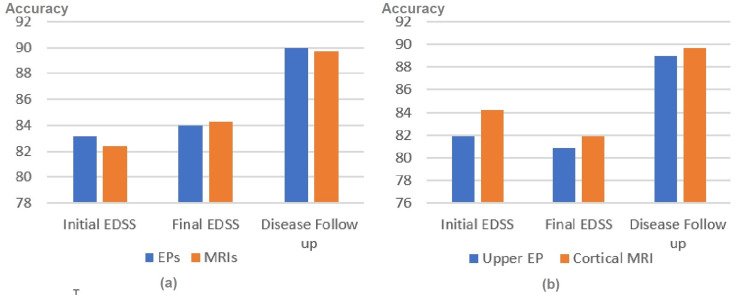
ML prediction accuracy for different biomarkers. (a) These results show the accuracy among EPs and MRIs. (b) These results show the accuracy between specific EPs, upper EPs, and cortical MRIs. MEPs, as well as cortical MRI, are the most successful predictors for MS patients, with comparable performances. ML, machine learning; EPs, evoked potentials; MEP, multimodal evoked potentials; EDSS, Expanded Disability Status Scale

All of the above results highlight the importance of EPs as predictors of EDSS scores for both the initial diagnosis and follow-up of MS patients, based on their accuracy performance. According to Table 3, the decision tree, NN, and Bayes models for EPs attained accuracies of 88.9%, 92.9%, and 88.2%, respectively, which are comparable to those of MRI, which attained accuracies of 88.2%, 96.0%, and 85.0%. EPs could, therefore, be considered alternative predictors of MS with comparable efficacy to MRI findings (Figure 2).

Nevertheless, MEPs should always be included when available, as they increase the total accuracy to 96.0% for final EDSS prediction and, consequently, disease follow-up (94.5%). More data are needed to validate these results.

## Discussion

In our research, we have demonstrated the predictive prognostic value of SEPs in MS patients by showing a positive association between SEP latencies and the EDSS [47]. Other studies suggest that SEP and MEPs may be more sensitive than clinical and MRI measures in detecting disease progression [48]. Given their cost-effectiveness and time-saving advantages, EPs could be the best alternative to MRI.

The correlation between SEPs and EDSS scores in MS patients is significant, indicating that SEPs can serve as predictive markers for disability progression. Various studies have demonstrated that abnormalities in SEPs correlate with EDSS scores, particularly in the early stages of the disease. Other previous studies have introduced this correlation strength between EPs and the EDSS index. A study found that median nerve SEPs recorded in MS patients showed a correlation between abnormal potentials and higher EDSS scores, suggesting that more severe disruptions in the somatosensory pathway are associated with greater disability [49]. In addition, the predictive value of early abnormalities in EPs has been linked to long-term disability outcomes, with significant correlations observed after five and 10 years. Thus, a predictive model combining MEPs and clinical variables provided accurate short-term disability estimates, with most EDSS predictions showing minimal error. According to clinical implications, these findings suggest that SEPs can help identify patients at risk for rapid disability progression, allowing for timely therapeutic interventions [50].

Additionally, AI could result in patient-specific prediction models for a better understanding of disease progression and personalized treatment planning. The predictive value of EPs in MS patients has been demonstrated in various studies, showing significant correlations between EP abnormalities and EDSS scores [51]. These findings suggest that EPs can serve as valuable predictive markers for disability progression, particularly in the early stages of the disease. The correlation between SEPs and EDSS scores has been established, with more severe disruptions in the somatosensory pathway associated with greater disability [52]. MEPs have also been found to correlate strongly with changes in EDSS over time. Early abnormalities in EPs have been linked to long-term disability outcomes, with significant correlations observed after five and 10 years [53]. A predictive model combining MEPS and clinical variables has been shown to provide accurate short-term disability estimates. These findings have important clinical implications, as they suggest that EPs can help identify patients at risk for rapid disability progression, allowing for timely therapeutic interventions. AI could be used to develop patient-specific prediction models for a better understanding of disease progression and personalized treatment planning. By incorporating AI into the analysis of EP data, healthcare professionals could gain more accurate and individualized insights into disease progression and treatment outcomes, ultimately improving patient care and outcomes in MS management. The EPs could be demonstrated as predictors of MS with comparable efficacy to that of MRI findings. Additional research is warranted to establish EPs, which are significantly less expensive, more mobile, and easier to administer than MRI, as equally effective as imaging or biochemical methodologies in serving as biomarkers for MS.

Integrating ML algorithms could enhance predictive accuracy by analyzing vast datasets, and identifying patterns that may not be immediately apparent to clinicians. This approach not only streamlines the decision-making process but also empowers clinicians to make informed choices based on real-time data analysis, fostering a more proactive approach to patient management. In addition, the use of predictive analytics can facilitate early intervention strategies, allowing for timely adjustments in treatment plans that align with the evolving needs of patients. Moreover, as these technologies continue to evolve, they hold the potential to revolutionize the way healthcare providers approach chronic conditions, leading to more personalized and effective treatment pathways.

Limitations

However, there are some limitations that should be considered when interpreting the findings. First, the dataset used is specific to Mexican Mestizo patients diagnosed with MS between 2006 and 2010, which may limit the generalizability of the results to other populations with different genetic, environmental, and cultural backgrounds. Additionally, the size of the dataset may be insufficient to encompass all potential variables influencing MS progression, potentially impacting the robustness of the predictive model. The reliance on historical patient data and recollections also raises concerns about bias and variability in reporting symptoms and progression. Furthermore, the ML algorithms employed assume independence among features, which might not always hold true and could lead to inaccuracies in predictions. Finally, the findings require validation in larger, multi-center studies to strengthen their conclusions and ensure applicability across diverse patient populations.

## Conclusions

In conclusion, our research shows that SEPs and MEPs can play a key role in predicting how MS progresses. We have found a strong link between SEP latencies and EDSS scores, which means these tests could be reliable markers for tracking the disease and predicting future disability. What makes EPs especially promising is that they are more affordable, easier to use, and quicker than traditional MRI scans. On top of that, using AI and ML to analyze EP data opens new doors. These technologies can boost the accuracy of predictions (96.0% for final EDSS prediction), help catch early signs of rapid disease progression, and support more personalized treatment plans. With AI-driven insights, doctors can make better, faster decisions, leading to improved outcomes and quality of life for MS patients.

Looking ahead, larger, long-term studies are needed to confirm how effective EPs are as standard markers for MS. It would also be valuable to explore how combining EPs with clinical data and imaging results can enhance these predictive models. As technology continues to evolve, it holds great potential to transform how we diagnose, predict, and manage MS, paving the way for more personalized and effective patient care.
